# Identification of a novel *TP63* mutation causing nonsyndromic cleft lip with or without cleft palate

**DOI:** 10.1186/s12920-021-00903-4

**Published:** 2021-02-23

**Authors:** Tianhui Xu, Mengmeng Du, Xinhua Bu, Donglan Yuan, Zhiping Gu, Pei Yu, Xuefang Li, Jiao Chen, Chunyan Jin

**Affiliations:** Department of Medical Genetics and Prenatal Diagnosis, Hospital Affiliated 5 to Nantong University (Taizhou People’s Hospital), Taizhou, Jiangsu China

**Keywords:** Cleft lip with or without cleft palate, Nonsyndromic cleft lip with or without cleft palate, *TP63*, Whole exome sequencing

## Abstract

**Background:**

Cleft lip with or without cleft palate (CL/P) is the most common craniofacial anomaly with a high incidence of live births. The specific pathogenesis of CL/P is still unclear, although plenty of studies have been conducted. Variations of tumor protein 63 (*TP63*) was reported to be related to the phenotype of CL/P. The case discussed in this report involves a pedigree with mutation at *TP63* gene, and the variation was not reported before.

**Case presentation:**

A Chinese pedigree with CL/P was collected in this study. The proband is a 3-year-old boy with the phenotype of CL/P, while his global development and intelligence are normal. After two CL/P repair operations, he looks almost normal. The proband's uncle and grandmother both have the phenotype of CL/P. Cytogenetic analysis and chromosomal microarray analysis (CMA) were performed, followed by whole exome sequencing (WES) and sanger validation. Analysis of WES revealed a variant of C>T at nucleotide position 1324 (1324C>T) of *TP63* gene, possibly producing a truncated protein with a premature stop codon at amino acid position 442 (p.Q442*). This mutation was localized at the oligomerization domain (OD) of *TP63* and might impair the capacity of p63 oligomerization.

**Conclusion:**

The mutation in *TP63* was recognized to be the possible cause of the phenotype of CL/P in this pedigree. This report provides some evidence for the clinical diagnosis of CL/P. And our study also provides clinical evidence for the molecular mechanism of *TP63* gene causing nonsyndromic cleft lip with or without cleft palate (NSCL/P).

## Background

CL/P is a serious congenital birth defect happened in about 1.7 per 1000 liveborn babies [1]. It has become one of the most common developmental malformations in the world. In addition to facial deformities, functions including mastication, swallowing, pronunciation and respiration of patients would be affected to varying degrees, and even psychological development could be abnormal. Approximately 70% of CL/P occur as an isolated abnormality which was called NSCL/P [2].

CL/P is a disorder caused by the combination of genes and environmental interactions. At present, a variety of genetic pathogenic factors of CL/P are identified, including chromosome 8q24.21, 17q22 and 10q25.3, *TGFα, TP63, IRF6, Runx2*, etc. through different methods such as CMA, WES and so on [3–8]. However, the specific pathogenesis of NSCL/P is still unclear. Among these reported genes, *TP63* is a key factor in the regulation of epithelial cell development, encoding a large number of *p63* subtypes. It has been reported that the variation of *TP63* could affect the binding ability of protein to DNA. Thomason et al. showed that the loss of *TP63* function led to the phenotype of CL/P in mice [9].

**Fig. 1:**
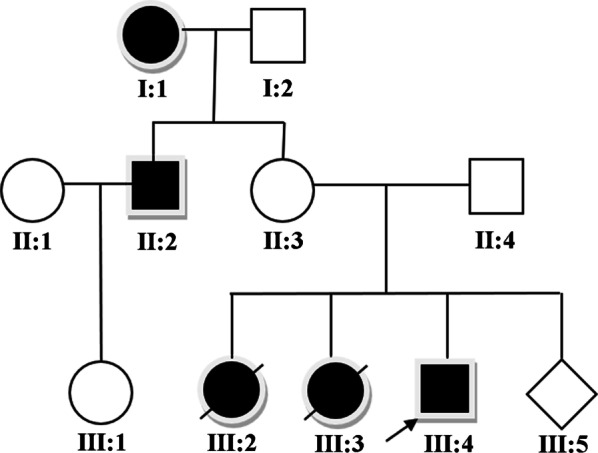
Pedigree of the family. I1: grandmother of the proband with phenotype of CL/P. II2: uncle of the proband with phenotype of CL/P. III4: the proband with the phenotype of CL/P. III2 and III3: aborted fetuses with the phenotype of CL/P. III5: fetus of the proband’s mother with no CL/P phenotype. I2, II1, II3, II4 and III1: members of the pedigree with no CL/P phenotype

In this study, we report the clinical findings detected in a Chinese pedigree (Fig. 1). Cytogenetic analysis and CMA of the pedigree members were normal. The proband was diagnosed by WES to be heterozygous for a C>T point mutation at nucleotide position 1324 (1324C>T) of *TP63*. The mutation was confirmed by sanger sequencing and it was not reported before.

## Case presentation

The proband is a 3-year-old boy with healthy parents. He was full-term vaginally delivered with a birth weight of 3450 g and a length of 50 cm. Ultrasound examination revealed the signs of cleft lip and cleft palate during pregnancy of the proband's mother. Repair of cleft lip was performed 4 months after birth and palatoplasty was performed at 18 months old. At present, the proband's development and intelligence are normal without other deformities. The mother of the proband had a history of two terminations of pregnancy because of cleft lip and cleft palate of the fetus. The proband's uncle and grandmother both have the phenotype of cleft lip and cleft palate.

**Fig. 2:**
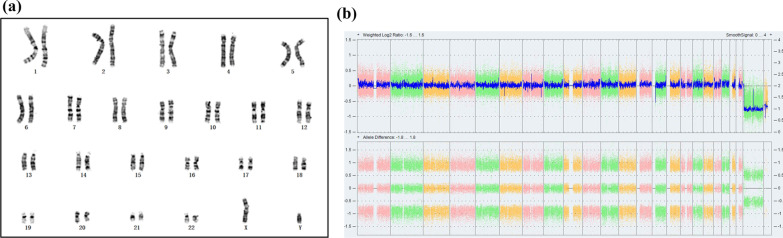
The results of cytogenetic analysis (**a**) and CMA (**b**) of the proband were normal

**Fig. 3:**
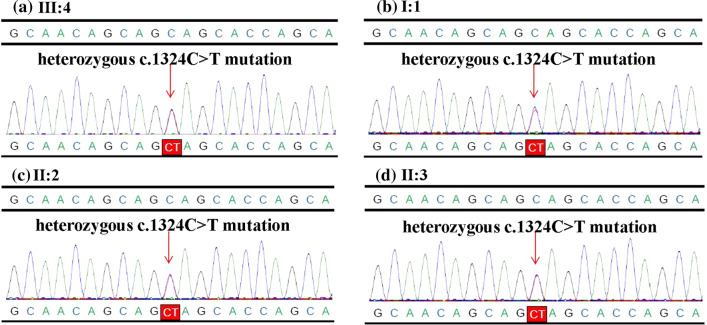
The mutation c.1324C>T in *TP63* gene of the proband (**a**), the grandmother (**b**), the uncle (**c**), and the mother (**d**) was detected by WES and confirmed by sanger sequencing

Cytogenetic analysis and CMA of the proband (Fig. 2) and other family members of the pedigree (data not shown) were normal. Results of WES analysis showed that the proband, the proband’s mother, uncle and grandmother carried the same c.1324C>T (p.Q442*) mutation in *TP63* gene, possibly producing a truncated protein (Table 1). However, this mutation was absent in other family members as shown in the pedigree (Fig. 1) as well as healthy population controls. This c.1324C>T (p.Q442*) mutation of *TP63* was not reported in literature before and also not found in NCBI dbSNP, genome AD, ExAC, and 1000 human genome dataset. Confirmation of Sanger sequencing was consistent with that of the WES (Fig. 3). In summary, this mutation can be interpreted as Likely Pathogenic according to ACMG guidelines [11].

**Table 1:** Genotype of the six family members

Name	Members of the pedigree	Gene	Exon / intron	Transcript number	Nucleotide change	Amino acid changes	Heterozygous / homozygous	Source of variation
IV:4	Proband	*TP63*	Exon10	NM_003722.5	c.1324C>T	p.Q442*	Heterozygous	III:3
III:2	Uncle	*TP63*	Exon10	NM_003722.5	c.1324C>T	p.Q442*	Heterozygous	II:4
III:3	Mother	*TP63*	Exon10	NM_003722.5	c.1324C>T	p.Q442*	Heterozygous	II:4
III:4	Father	/	/	/	/	/	Not detected	/
II:4	Grandmother	*TP63*	Exon10	NM_003722.5	c.1324C>T	p.Q442*	Heterozygous	/
II:5	Grandfather	/	/	/	/	/	Not detected	/

## Discussion and conclusion

All patients with the phenotype of CL/P had the same mutation site of *TP63* in this pedigree. It has been reported that mutations in *TP63* underlie several monogenic malformation syndromes manifesting CL/P including Rapp-Hodgkin syndrome, ankyloblepharon-ectodermal dysplasia-cleft lip/palate syndrome, ectrodactyly-ectodermal dysplasia-cleft lip/palate syndrome, limb-mammary syndrome, and acro-dermato-ungual-lacrimal-tooth syndrome [12]. Leoyklang et al. investigated the possibility of *TP63* mutation causing NSCL/P [2]. Variation of *TP63* was revealed to affect the binding ability of protein to DNA. Animal experiments in mice have found that loss of *TP63* function led to the phenotype of CL/P [9]. Study of Khandelwal et al. revealed that 6 of *TP63* heterozygous variants detected in CL/P patients were inherited from an unaffected parent, suggesting reduced penetrance of such loss-of-function alleles. Incomplete penetrance has also been observed for other *TP63* variants, including common variants such as those affecting arginine 319 (280accordingtoformernomenclature) [13]. Therefore, it explained why the mother of the proband did not show the phenotype of CL/P, although she carried the same mutation in *TP63.* Clinical variability and reduced penetrance are typical for *TP63* variants.

**Fig. 4:**
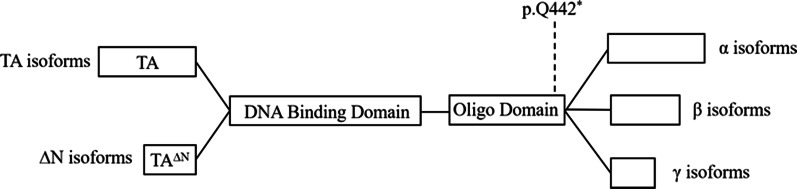
TA- and DN-p63 isoforms derived from alternative promoters

*TP63* plays an important role in the regulation of limb, epithelial, and craniofacial development [14]. This gene has at least six different isoforms, three of which containing the transactivating (TA) domain (TA isotypes) and the other three, none (△N isotypes). Alternative splicing at the 3′ end of the gene results in mainly three different C-terminal ends (α, β and γ). All isoforms contain a DNA-binding domain (DBD) and an OD, differing in the TA domain, sterilealpha-motif domain (SAM), auto-regulatory TA inhibiting domain (TI domain), and a second TA domain (TA2 domain) [15]. Kantaputra et al. implied that mutation in SAM domain of *TP63* is associated with NSCL/P [16]. Khandelwal’s study indicated that missense variants affecting the OD might impair the capacity of p63 oligomerization [13]. The 1324C>T mutation of *TP63* in the pedigree is a nonsense mutation. The amino acid at 442 of *TP63* was changed from glutamine to a termination codon (p.Q442 *), leading to the premature termination of peptide chain synthesis. *TP63* mutation discussed in this report was localized in the OD (Fig. 4). This variant may abrogate the oligomerization of mutant p63 protein into oligomeric complexes, resulting in the phenotype of CL/P. Therefore, c.1324C>T variant of *TP63* likely represents a loss-of-function allele rather than a dominant-negative pattern [13]. The frequency of the mutation was not included in the NCBI dbSNP, genome AD, ExAC and 1000 Genomes databases. We speculate that the mutations of the *TP63* gene (c.1324C>T) found in the Chinese family may be responsible for the phenotype of NSCL/P.

Although a variety of mutations in *TP63* have been reported in patients with SCL/P or NSCL/P, in-depth studies are needed to explore the specific pathogenesis. Leoyklang et al. recognized that a mutation in *TP63* causing NSCL/P may occur at a site different from those underlying mendelian malformation syndromes. The phenotypic differences depend on variability of *TP63* expression (2). This report demonstrates that this novel 1324C>T mutation is associated with NSCL/P, which highlights the wide phenotypic spectrum of *TP63* mutations.

Our study not only helps to reveal the pathogenic gene of the pedigree but also provides the research basis for the molecular mechanism of *TP63* gene causing the phenotype of NSCL/P.

## Data Availability

The datasets generated during the current study are available in the NCBI Sequence Read Archive (SRA) database (accession number: PRJNA701188), direct link: https://www.ncbi.nlm.nih.gov/sra/PRJNA701188.

## Abbreviations

CL/P: Cleft lip with or without cleft palate
CMA: Chromosomal microarray analysis
WES: Whole exome sequencing
